# Sonochemical Synthesis of Silver Nanoparticles Using Starch: A Comparison

**DOI:** 10.1155/2014/784268

**Published:** 2014-01-22

**Authors:** Brajesh Kumar, Kumari Smita, Luis Cumbal, Alexis Debut, Ravinandan Nath Pathak

**Affiliations:** ^1^Centro de Nanociencia y Nanotecnologia, Universidad de las Fuerzas Armadas (ESPE), Sangolqui, Ecuador; ^2^Department of Chemistry, Kolhan University, Chaibasa, Jharkhand 833202, India

## Abstract

A novel approach was applied to synthesize silver nanoparticles using starch under sonication. Colloidal silver nanoparticles solution exhibited an increase of absorption from 420 to 440 nm with increase starch quantity. Transmission electron microscopy followed by selected area electron diffraction pattern analysis indicated the formation of spherical, polydispersed, amorphous, silver nanoparticles of diameter ranging from 23 to 97 nm with mean particle size of 45.6 nm. Selected area electron diffraction (SAED) confirmed partial crystalline and amorphous nature of silver nanoparticles. Silver nanoparticles synthesized in this manner can be used for synthesis of 2-aryl substituted benzimidazoles which have numerous biomedical applications. The optimized reaction conditions include 10 ml of 1 mM AgNO_3_, 25 mg starch, 11 pH range, and sonication for 20 min at room temperature.

## 1. Introduction

Nanoscale materials have received considerable attention because their unusual optical, chemical, photoelectrochemical, and electronic properties differ significantly from those of atoms and molecules as well as those of bulk materials [1–3]. The synthesis of nanomaterials with the desired quality is one of the most exciting aspects in modern nanoscience and nanotechnology [4]. Colloidal nanoparticles are small in diameter but large in surface area and huge in current many exclusive medical and industrial applications such as biological engineering, catalysts, and electronic devices [5]. Colloidal silver nanoparticle (Ag-NP) with natural macromolecule can be fabricated by physical [6, 7] and chemical reduction [8–10] methods. Nowadays, biomass starch as a raw material for the synthesis of Ag-Nps has reflected significant superiority in some process. However, no literature is available on its preparation in starch at different pH and polysaccharide by ultrasonic field. The sonochemical methods are as follows: formation, development, and the implosion of the microcavities [11]. When solutions are exposed to ultrasonic irradiation, bubbles in the solution could be imploded by acoustic fields. Cavitation's bubble collapse can also induce a shock wave in the solution and drive the rapid impact of the liquid to the surface of the particles [12]. Use of the sonoelectrochemical method for the preparation of spheres, rods, and dendrites shaped Ag-Nps with nitriloacetate (NTA) [13]. It was found that the electrolyte composition that comes along reaction time can greatly affect the shape and growth of the NPs. Branched silver structure with *λ*_max⁡_ 440 nm and average diameter of 11.5 nm was formed using an aqueous extract of* Mesua ferrea* Linn. leaf [14]. Nagata and coworkers formed stable colloidal dispersions of silver prepared by ultrasonic irradiation of aqueous AgNO_3_ or AgClO_4_ solutions in the presence of surfactants [15]. Amorphous Ag-NPs of 20 nm size were prepared from an aqueous solution of AgNO_3_ using argon-hydrogen atmosphere [16] and AgBr in the presence of gelatin [17]. Sonochemical route using a hazardous reducing agent (NaBH_4_) produced spherical silver nanoparticles of sizes 10 nm [18].

Many methods aiming at the formation of Ag-Nps, including the green ones, make use of an organic molecule. The latter interacts with the particles and provides them with stability against oxidation and agglomeration, or it can even act as a matrix only. In this sense, polymer molecules have been widely employed because their long chain offers many binding sites in which nanoparticles can be stabilized [19]. Moreover, natural polymers are extremely important because many of them are biocompatible and nontoxic. Among such biomolecules are sucrose [20], maltose [20], chitosan [21], Arabic gum [22], and plant extracts such as the ones obtained from *Jatropha curcas* [23], *Murraya koenigii*, [24] and *Mangifera indica* [25]. More specifically, the natural rubber latex (NRL) extracted from *Hevea brasiliensis*, a native tree from the Amazon forest, arises as a possible biomaterial for use in the synthesis of nanoparticles [26].

The use of green capping and stabilizing attribute of starch in aqueous solution has recently become important in synthesis methods of nanomaterials, due to the fact that this biopolymer acts as an effective surfactant agent and is environmentally friendly. It is possible to obtain Ag-NPs of spherical shape by using sago starch as coating agent [27]. Shervani et al. [28] synthesized silver NPs of 15 and 43 nm using 1 wt% aqueous solution of starch. Others studies [29, 30] found that stable Ag-NPs have could been synthesized by using soluble starch both as reducing and stabilizing agents, with sizes in the range of 10–34 nm.

**Figure 1:**
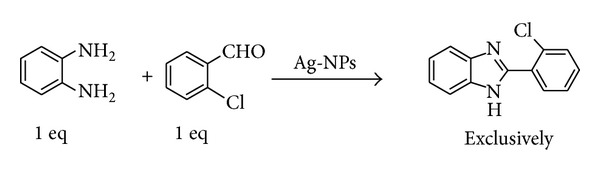
Schematic scheme for synthesis of 2-(2-chlorophenyl)-1*H*-benzimidazole.

In this paper, we report a green approach toward the sonochemical synthesis and stabilization of Ag-Nps by using starch; and its application as a catalyst for synthesis 2-(2-chlorophenyl)-1*H*-benzimidazole (Figure 1).

## 2. Material and Methods

### 2.1. Chemicals

All chemicals used were of analytical grade and used without any purification. Silver nitrate (99.0%) was purchased from Spectrum (USA). Acetonitrile, ethyl acetate, hexane, substituted benzaldehyde, and *o-*phenylenediamine were purchased from Thomas Baker (INDIA). Millipore Milli-Q water was used in all experiments.

### 2.2. Synthesis and Characterization of Starched Ag-Nps

Preparation of starched Ag-Nps is quite straight forward. In a typical preparation, 25 and 30 mg of starch was added to 10 mL of 1 mM of AgNO_3_. The mixture was stirred for complete dissolution and agitated under sonication. Ultrasound irradiation was carried out with ultrasonic processors (DAIGGER GE 505, 500 W, 20 kHz) immersed directly into the reaction solution. The operating condition was at 30 sec pulse on and 30 sec pulse off time with amplitude of 72% at 25°C for 20 minutes. The mixture was prepared and observed at different pH (5.5−11) under sonication. pH measurements of silver nitrate solution were done by using pH meter (Seven Easy pH, METTLER TOLEDO AG, 8603, Switzerland). The reduction of silver ions was monitored at intervals of hours and days, followed by measurement of the UV-Vis spectra using spectrophotometer (Thermo Spectronic, GENESYS 8, England, Quartz Cell, path length 10 mm and Graph Plotted on Origin 6.1 program). To find the highest peak or *λ*_max⁡_, a spectral analysis was carried out by measuring the optical density of the content from wavelength 200 to 800 nm. The particle size distributions of nanoparticles were determined using the HORIBA, Dynamic Light Scattering Version LB-550 program. Size and diffraction pattern of nanoparticles are studied on transmission electron microscopy, TEM (FEI, TECNAI, G^2^ spirit twin, Holland). A thin film of the sample was prepared on a carbon-coated copper grid by dropping a very small amount of the sample on the grid. Fourier transform infrared (FTIR-ATR) spectra were recorded on a PerkinElmer (Spectrum two) spectrophotometer to hypothesized functional groups involved in the synthesis of starches Ag-Nps. X-ray diffraction (XRD) studies on thin films of the nanoparticle were carried out using a BRUKER D8 ADVANCE brand *θ*-2*θ* configuration (generator-detector) X-ray tube copper *λ* = 1.54 Å and LYNXEYE PSD detector. The diffracted intensities were recorded from 20° to 70° 2*θ* angles.

### 2.3. Synthesis and Characterization of 2-(2-Chlorophenyl)-1*H*-benzimidazole

A mixture of 1,2-phenylenediamine (1.0 mmol) and *o-*chlorobenzaldehyde (1.0 mmol) in acetonitrile (3.0 mL) was taken, and 100 *μ*L of Ag-Nps was added at room temperature. The reaction solution was stirred at room temperature for 10 minutes. The reaction was monitored until its completion by thin layer chromatography (TLC). The filtrate was evaporated under vacuum and subsequently dried to afford the crude product which was purified by column chromatography using hexane/ethyl acetate as eluent to afford pure benzimidazole (approx. 95%). The structure was characterized by NMR, ESI-MS, and melting point. ^1^H NMR spectra of CDCl_3_ solutions were obtained with an Advance 500 (Model: Bruker, 11.4 Tesla, 500 MHz) spectrometer using tetramethylsilane (TMS) as the internal standard. ESI-MS were obtained on a Varian 91 500-LC ion trap mass spectrometer. Melting points determined on a digital Stuart SMP 10 melting point apparatus (ST15, OSA, UK) are uncorrected. The thin layer chromatography (TLC) was performed using the aluminum sheets coated with silica gel 60 (MERCK) containing fluorescent indicators, F254.

## 3. Results and Discussion

### 3.1. Comparative Study of Carboxymethyl Cellulose, Starch, and Sucrose under Sonication at pH 8.5

**Figure 2:**
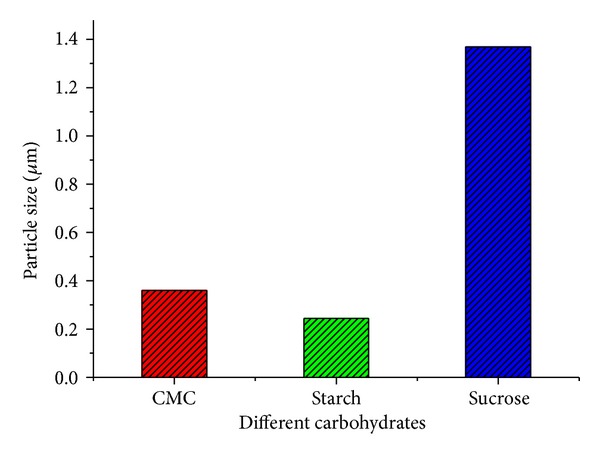
Size of Ag-Nps synthesized by using different carbohydrates at pH 8.5.

The bonding nature of carbohydrate plays an important role in reduction of silver ion into Ag-Nps. So, it is very important deciding factor for choosing bioreductant. We started our experiments as hit and trial method with carboxymethyl cellulose (CMC), starch, and sucrose at pH 8.5 under sonication. Addition of 25 mg of CMC (90,000 MW), starch, and sucrose to the 1 mM AgNO3 solution, the reaction completed by 20 min with change of light yellow color. Particle size analyzer (DLS) used to examine the size of controlled nanoparticles in aqueous suspensions.  Figure 2 shows the comparative graph of size of Ag-Nps which was recorded after the completion of the reaction. We observed that starch (0.2437 *μ*m) has given better result in comparison to CMC (0.3595 *μ*m) and sucrose (1.369 *μ*m). The cause behind this observation was that starch is polysaccharide that has *α*-glycosidic bond (which is a covalent bond between two monosaccharides that involves the carbon C1 anomeric of the first unit of glucose and carbon C4 of the second unit of glucose) which cleaved under basic medium and sonication to give free aldehyde group. So, it acts as reducing sugar and reduces Ag+ to Ag-Nps and gluconic acid [10] whereas CMC has also o-glycosidic but less tendency to reduce due to its high cross-linking structure [31]. Sucrose is a disaccharide consisting of one unit of *α*-glucose and one unit of *β*-fructose linked by *β*-glycosidic bonds, which is a covalent bond between two monosaccharides that involves carbon C1 (anomeric) of the glucose and carbon C2 of the fructose. So it has less tendency due to its nonreducing *β*-glycosidic bond [20]. On looking this reducing and stabilizing nature of starch, we started our further experiment only with starch.

### 3.2. Effect of pH on Particle Size

**Figure 3:**
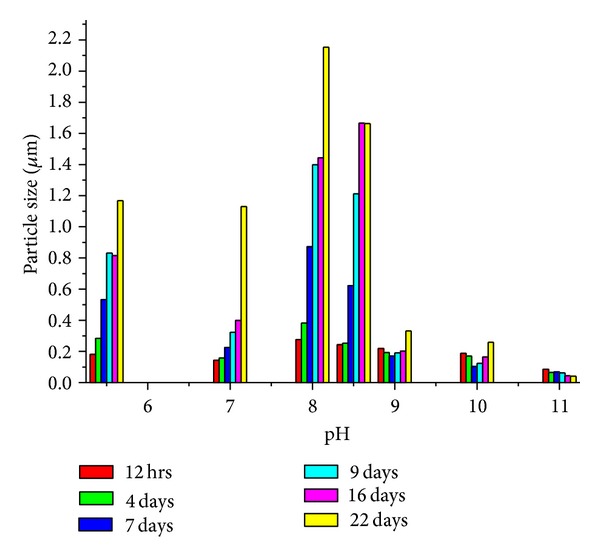
Effect of pH on particle size at different time duration.

The pH of 10 mL, 1 mM silver nitrate was adjusted to 5.5, 7, 8, 8.5, 9, 10, and 11 using dilute sodium hydroxide (0.1 N); 25 mg starch was added with continuous stirring. After complete addition, the reaction was allowed to proceed under sonication for 20 mins whereby silver colloid was formed (Table 1). It is important to note that we performed the reaction up to pH 11 because the very high alkaline condition of the medium may lead to weakening of the association (H-bonding) even for the starch matrix and thereby diminishing its templating potential to support small sized nanoparticles [32]. A further increase in the basicity of the medium may be resulting in a red shift of the peak position.  Figure 3 shows the respective graph of particle size (DLS) of the silver colloid obtained using starch as reducing and stabilizing agent at different pHs. The results reveal a number of observations which may be summarized as follows: (a) increasing the pH of the solution from 5.5 to 8.0 is accompanied by increase of particle size and pH of the solution from 8.0 to 11.0 abrupt decrease of particle size; (b) the smallest particle size was observed at pH 11 and the size of Ag-Nps decreases with time from 4 hrs to 22 days (0.0857 to 0.0411 *μ*m); (c) particle size continuously increases with time from pH 5.5 to 8.5 in the reaction mixture; (d) the particle size decreases from 12 hrs to 9 days and then increases from 9 days to 22 days at pH 9 and 10; (e) when the pH 11 is targeted, the size becomes smaller and could be assigned to the Ag-Nps.

### 3.3. Effect of pH on UV-Visible Absorbance

**Figure 4:**
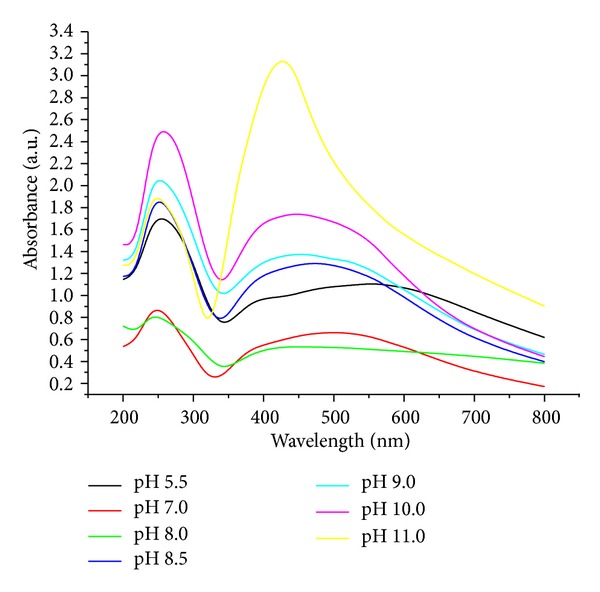
UV-Vis spectra at different pH after 4 days.

Figure 4 shows the UV-Vis spectra of the silver colloid obtained using starch as reducing and stabilizing agent at different pHs. In acidic pH, the association between the functional groups of starch through H-bonding is expected to be more pronounced, which stabilizes the structures and as a result of which these compounds are not effectively utilized in the reduction process. This feature is reversed with the increase in basicity. With the increase of pH up to 11, the intensity of the peak at 420 nm kept on increasing which indicates the progressive generation of the nanoparticles. The results reveal a number of observations which may be summarized as follows: (a) increasing the pH of the solution is accompanied by appreciable changes in the electronic absorption spectra; (b) a band at higher energy, that is, 240 nm, appears at pH 10 and the intensity of this band decreases by decreasing the pH up to 5.5; (c) further decreases in the pH of the reaction medium up to pH 11 lead to lowering intensity of this band; (d) simultaneously, another band at 420 nm starts to appear and the absorption peak that appeared is attributed to the surface plasmon excitation of silver particles and reaches its maximum intensity at pH 11; (e) when the range of pH 11 is targeted, the band becomes stronger and could be assigned to the plasmon resonance of Ag-Nps.

### 3.4. Effect of Starch on Particle Size

#### 3.4.1. UV-Visible and DLS Analysis of Ag-Nps

**Figure 5:**
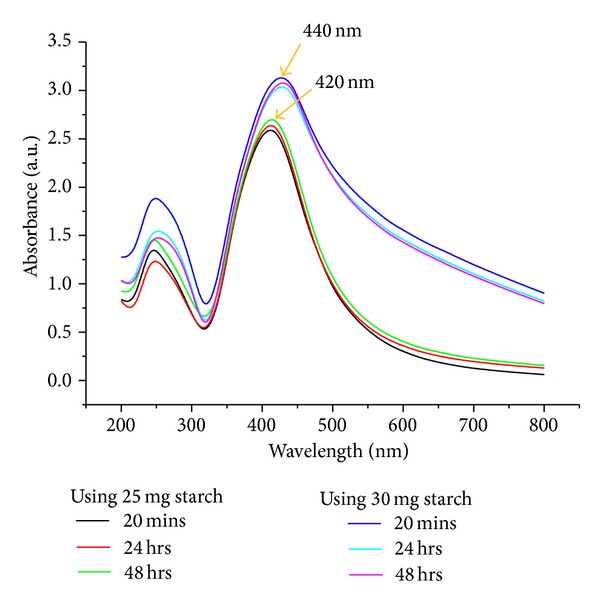
UV-Vis spectra of Ag-Nps at different time at pH 11.

**Figure 6:**
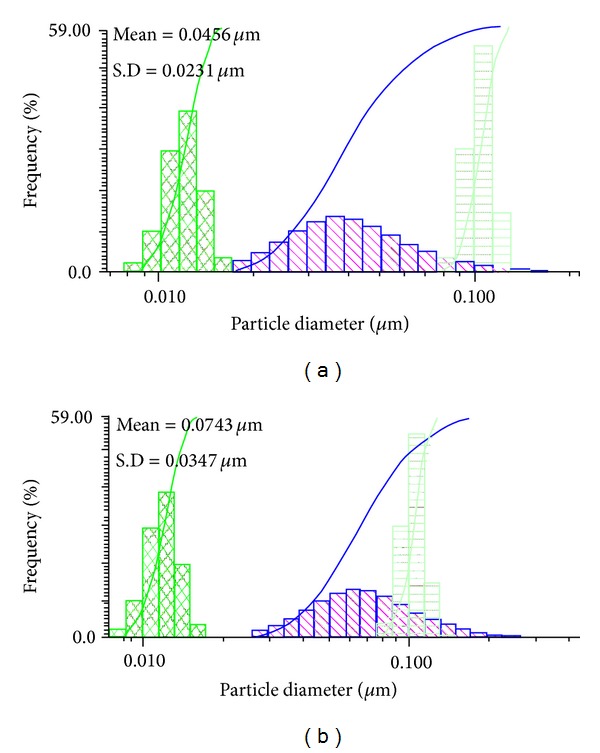
DLS image of Ag-NPs synthesized under ultrasonic irradiation for 20 min, after 4 days: (a) 25 mg and (b) 30 mg starch.

Figure 5 shows the UV-Vis absorption spectra of silver nanoparticles colloidal solutions prepared at different amounts of starch (25 and 30 mg) at pH 11. The data reveal several important findings which can be presented as follows: (i) using 25 and 30 mg of starch with 1 mM AgNO_3_ reaction under 20 min sonication leads to outstanding enhancement in the plasmon intensity indicating that large amounts of silver ions are reduced and used for cluster formation; (ii) keeping both reactions (25 and 30 mg starch) from 20 min to 48 hrs is accompanied by marginal increment in the absorption intensity which could be attributed to stability of Ag-Nps; (iii) the absorption intensity observed at 420 nm and 440 nm reveals some aggregation of Ag-Nps at 30 mg starch; (iv) increasing the reaction duration up to 48 hrs, the band becomes stronger and could be assigned to the plasmon resonance of Ag-Nps at 420 and 440 nm.  Figure 6 shows the size distribution of 25 and 30 mg starched Ag-Nps; mean and standard deviation for 25 mg starched Ag-Nps was 0.0456 and 0.0231 *μ*m whereas for 30 mg starched Ag-Nps was 0.0743 and 0.0231 *μ*m. DLS study reveals that the size of 25 mg starched Ag-Nps is smaller than 30 mg; its value also agrees with the interpretation UV-Vis data (220 and 240 nm) due to aggregation of starched Ag-Nps at higher concentration.

#### 3.4.2. TEM and SAED Analyses of Ag-Nps

**Figure 7:**
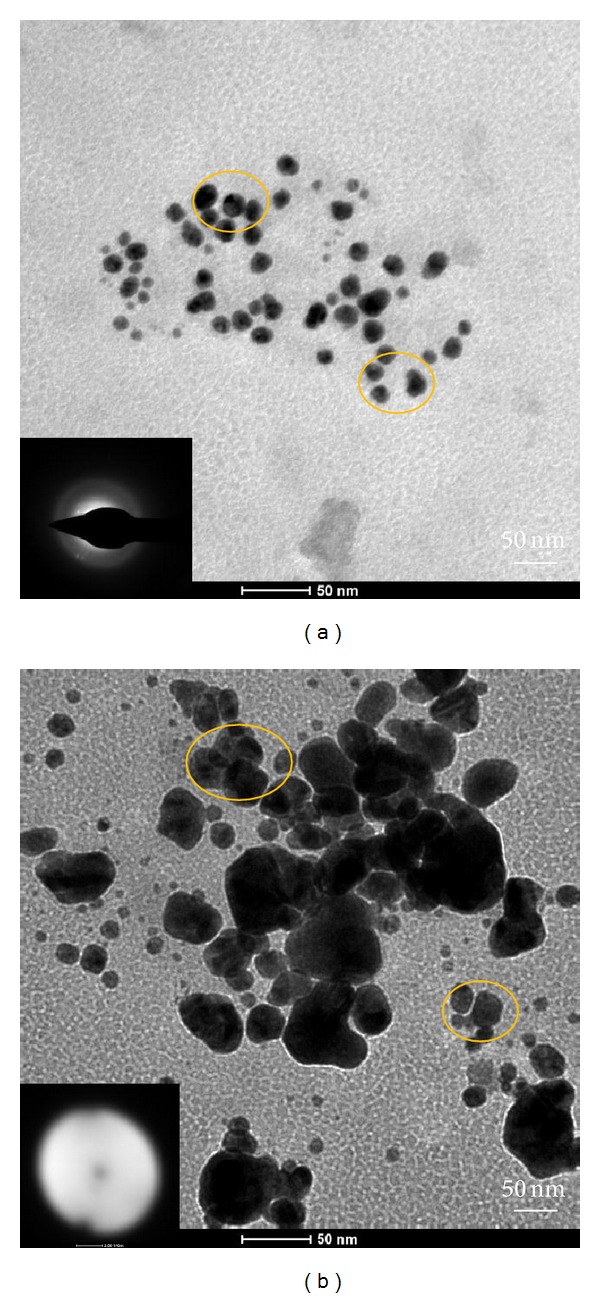
TEM and SAED pictures of Ag-Nps: (a) 25 mg starch and (b) 30 mg starch.

Figure 7 shows the TEM images of Ag-Nps formed after 4 days. TEM image shows small spherical shape and polydispersed particles. The corresponding average size distribution for 25 mg starched Ag-Nps is 10 to 25 nm whereas 30 mg starched Ag-Nps are 23 to 97 nm. The Ag-Nps are spherical in shape, selected area electron diffraction pattern explains their partial crystalline nature, and are not aggregated in solution with 25 mg starch for 20 min sonication (Figure 7(a)). This is due to the binding force between the Ag-NPs and the capping molecules that may get decreased whereas 30 mg starched Ag-Nps were aggregated. The diffuse band in the electron diffraction pattern is due to the presence of starch and explains amorphous nature Ag-Nps (Figure 7(b)). The smallest size of the synthesized Ag-Nps is 10 nm and 23 nm by using 25 and 30 mg starch, respectively.

#### 3.4.3. FTIR Analysis of Ag-Nps

**Figure 8:**
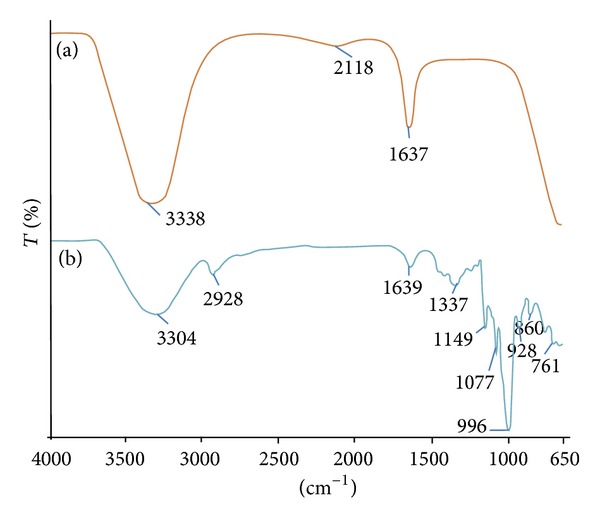
FT-IR spectrum (a) synthesized Ag-Nps (b) Starch.

FT-IR measurements were carried out to identify the possible functional group responsible for the reduction of the Ag^+^ ions and capping of the bioreduced Ag-Nps synthesized by starch.  Figure 8(b) represents the FTIR spectrum of starch and shows bands at 3304 cm^−1^ for –OH stretching (aliphatic hydroxyl group), 2928 cm^−1^ for C–H stretching (aliphatic C–H), and 1639 cm^−1^ for carbonyl stretching (C=O, carbonyl group). In Figure 8(a), there is deviation of both hydroxyl and carbonyl group; it appears at 3338 and 1637 cm^−1^, indicating that both were involved in synthesis of Ag-Nps.

#### 3.4.4. XRD Analysis of Ag-Nps

**Figure 9:**
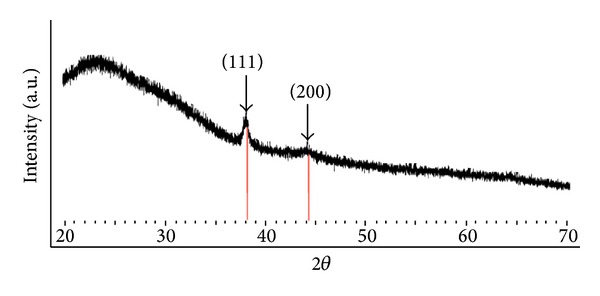
X-ray diffraction pattern of starch impregnated with Ag-NPs.

In the XRD spectrum (Figure 9), the broad reflexion at 20° to 30° is due to the low crystallinity of the starch, and other peaks are assigned to diffractions from the (111) and (200) planes of face-centred cubic (fcc) silver (Ref. no-01-087-0717). SAED pattern of AgNPs (Figure 7) also showed a diffuse band corresponding to the polymorph of starch. Because the diffraction pattern of silver nanoparticles could not be observed, it indirectly explains the amorphous nature of AgNPs. We hypothesized that an extensive number of hydroxyl groups can facilitate the complexation of metal ions to the molecular starch matrix. Ag^+^ reduced to Ag and Ag may be completely wrapped or be inside the starch molecule. XRD and SAED pattern indicates the partial amorphous nature of starched AgNPs.

#### 3.4.5. NMR-ESI MS Analysis of 2-(2-Chlorophenyl)-1*H*-benzimidazole [33]

**Figure 10:**
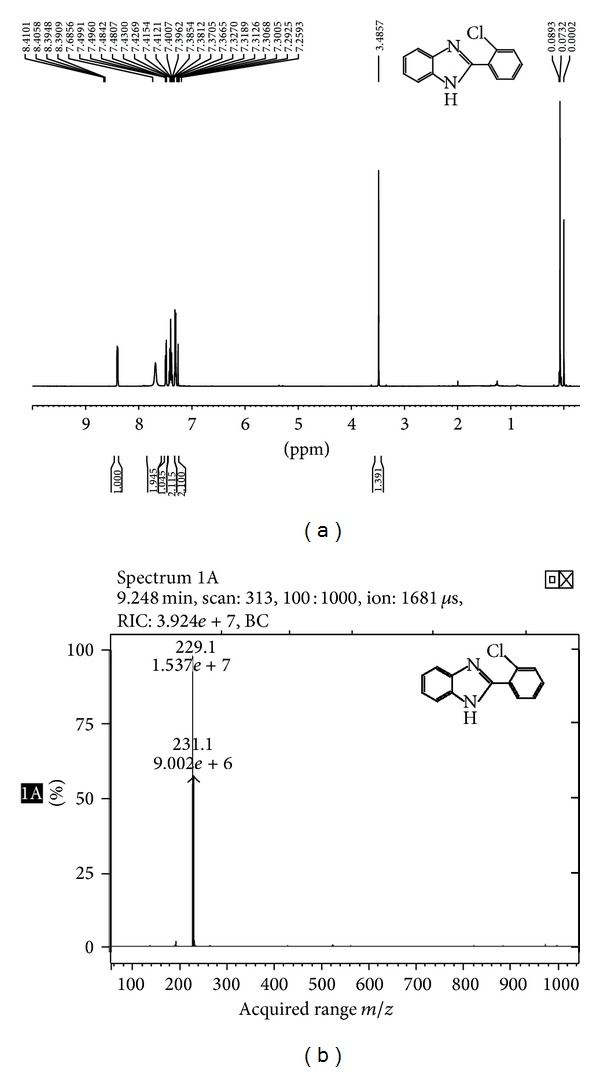
(a) ^1^H NMR and (b) ESI-MS spectra of 2-(2-chlorophenyl)-1*H*-benzimidazole.

We used starched Ag-Nps as catalyst for synthesis of 2-aryl benzimidazole derivatives. In our initial experiments, we choose 1,2-phenylenediamine (1.0 mmol) and *o-*chlorobenzaldehyde(1.0 mmol) as a model reaction for optimization of catalyst and reaction conditions. Figures 10(a) and 10(b) show ^1^HNMR and ESI-MS spectra of 2-(2-chlorophenyl)-1*H*-benzimidazole. These results confirmed that Ag-Nps significantly increased the selectivity for synthesis 2-aryl benzimidazole.

Chemical formula: C_13_H_9_ClN_2_; white solid (~95%); mp 230–232°C; ^1^H NMR (500 MHz, CDCl_3_): *δ* 8.40 (dd, 1H), 7.68 (br, 2H), 7.48 (m, 1H), 7.40 (m, 2H), and 7.31 (m, 2H); ESI-MS: *m*/*z* = 229.1 (M + 1).

#### 3.4.6. Mechanism for Ag-NP Catalyzed Synthesis of 2-Aryl Benzimidazole

**Figure 11:**
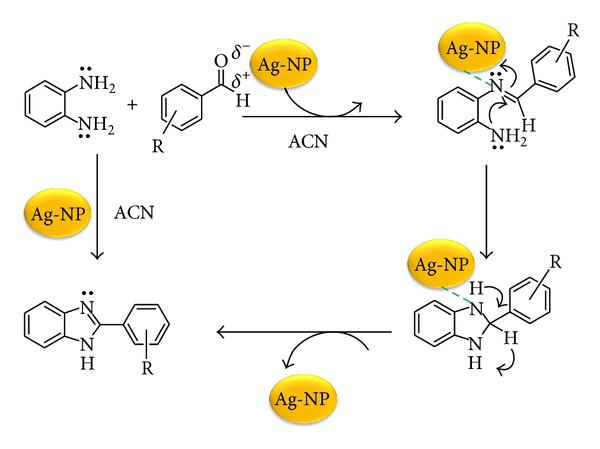
Generalized mechanism for Ag-NP catalyzed synthesis of 2-aryl benzimidazole.

We inspired from oxidative properties of Ag-NPs well documented by Cong et al. for the Diels-Alder cycloadditions of 2′-hydroxychalcones [34]. Based on our experimental results we propose the brief working mechanism generated by the AgNP's for the synthesis of 2-aryl benzimidazole derivatives as shown in Figure 11. The existence of electron transfer processes between the AgNP and an aldehyde substrate, consistent with the electron transfer processes in metal nanoparticle-catalyzed oxidation reactions [35–38].

## 4. Conclusions

In summary, we have demonstrated a simple, clean, effective, economical, nontoxic, and environment friendly technique for the synthesis of colloidal silver nanoparticle by using starch as reducing and stabilizing agent under sonication. Sonochemical reduction route demonstrates a remarkable potential for fabricating desired particle size colloidal silver nanoparticles. Absorption spectra confirm the presence of surface plasmon resonance at 420 and 440 nm, characteristic of Ag-Nps. A diverse range of pH was used to standardize the reaction at room temperature. In addition, the use of sonication for synthesis of Ag-Nps is more environmentally friendly and safer than other synthetic methodology. Starched Ag-NPs showed efficient catalytic activity for the synthesis of 2-aryl benzimidazole.

## Figures and Tables

**Table 1:** Average size of sonochemical starched Ag-NPs at different pH.

AgNO_3_/starch (1 mM)	pH	Sonication time	UV-visible absorption (nm)	Average particle size (µm)
10 mL/25 mg	5.5	20 min	560	0.1811
10 mL/25 mg	7.0	20 min	500	0.1424
10 mL/25 mg	8.0	20 min	440	0.2757
10 mL/25 mg	8.5	20 min	480	0.2558
10 mL/25 mg	9.0	20 min	460	0.2192
10 mL/25 mg	10.0	20 min	440	0.1876
10 mL/25 mg	11.0	20 min	420	0.0857
10 mL/30 mg	11.0	20 min	440	0.0978
